# Inducible knock-out of BCL6 in lymphoma cells results in tumor stasis

**DOI:** 10.18632/oncotarget.27506

**Published:** 2020-03-03

**Authors:** Stefanie Schlager, Carina Salomon, Sabine Olt, Christoph Albrecht, Anja Ebert, Oliver Bergner, Johannes Wachter, Francesca Trapani, Daniel Gerlach, Tilman Voss, Anna Traunbauer, Julian Jude, Matthias Hinterndorfer, Martina Minnich, Norbert Schweifer, Sophia M. Blake, Vittoria Zinzalla, Barbara Drobits, Darryl B. McConnell, Norbert Kraut, Mark Pearson, Johannes Zuber, Manfred Koegl

**Affiliations:** ^1^Boehringer Ingelheim RCV GmbH & Co KG, Vienna, Austria; ^2^Research Institute of Molecular Pathology (IMP), Vienna BioCenter (VBC), Vienna, Austria; ^3^Current address: AstraZeneca AB, Gothenburg, Sweden; ^4^Medical University of Vienna, Vienna BioCenter (VBC), Vienna, Austria

**Keywords:** BCL6, DLBCL, lymphoma, inducible CRISPR/Cas9, *in vivo* xenograft

## Abstract

Diffuse large B-cell lymphoma (DLBCL) is the most common type of non-Hodgkin lymphomas worldwide and is characterized by a high diversity of genetic and molecular alterations. Chromosomal translocations and mutations leading to deregulated expression of the transcriptional repressor BCL6 occur in a significant fraction of DLBCL patients. An oncogenic role of BCL6 in the initiation of DLBCL has been shown as the constitutive expression of BCL6 in mice recapitulates the pathogenesis of human DLBCL. However, the role of BCL6 in tumor maintenance remains poorly investigated due to the absence of suitable genetic models and limitations of pharmacological inhibitors. Here, we have utilized tetracycline-inducible CRISPR/Cas9 mutagenesis to study the consequences of BCL6 deletion in established DLBCL models in culture and *in vivo*. We show that BCL6 knock-out in SU-DHL-4 cells *in vitro* results in an anti-proliferative response 4–7 days after Cas9 induction that was characterized by cell cycle (G1) arrest. Conditional BCL6 deletion in established DLBCL tumors *in vivo* induced a significant tumor growth inhibition with initial tumor stasis followed by slow tumor growth kinetics. Our findings support a role of BCL6 in the maintenance of lymphoma growth and showcase the utility of inducible CRISPR/Cas9 systems for probing oncogene addiction.

## INTRODUCTION

DLBCL is an aggressive and genetically diverse B-cell neoplasm in adults resulting in a biologically and clinically heterogeneous disease. Standard of care treatment, which includes a combination of chemotherapy and the monoclonal CD20 antibody rituximab (R-CHOP), results in an initial response but ultimately leads to disease recurrence in 30% of patients for whom there remains a high unmet medical need [1].

Recent comprehensive sequencing studies in a large cohort of DLBCL patients highlight the heterogeneity of alterations including somatic mutations, copy number alterations, and structural variants [2–4]. Among the most frequently rearranged genes are IGH, BCL2, BCL6, and MYC, with 40%, 21%, 19%, and 8% of cases affected, respectively [5–8]. BCL6 is a DNA-binding protein that represses gene transcription in Germinal Center (GC) B-cells through the recruitment of co-repressor proteins. In GCs, BCL6 inhibits DNA damage response pathways and thereby prevents cell cycle arrest and apoptosis during class switch recombination and somatic hypermutation required for antibody maturation in B-cells. Subsequent BCL6 downregulation is crucial for differentiation into mature antibody-producing plasma and memory B-cells [9]. In a significant subset of lymphoid malignancies chromosomal translocations and mutations lead to BCL6 deregulation. Such genetic alterations include translocations that fuse its coding sequence to heterologous promoters [10], point mutations in BCL6 promoter negative regulatory elements [11, 12] or mutations that affect BCL6 transcription [13], acetylation-mediated BCL6 inactivation [14] or BCL6 degradation [15].

Constitutive BCL6 expression within GC B-cells leads to the development of DLBCL in mice that mimics that observed in patients [16, 17] suggesting that BCL6 is sufficient to initiate cancer. However, it remains not fully investigated whether BCL6 is relevant for tumor maintenance. A variety of BCL6 inhibitors have been previously reported, several of which have demonstrated that the BTB domain of BCL6 is amenable to targeting with peptide and small molecule inhibitors (reviewed in [18]) as well as PROTACs [19]. The BTB domain is required for interaction with co-repressor complex proteins to mediate transcriptional repression [20, 21]. Treatments with compounds that disrupt the interaction between BCL6 and the co-repressor complex have been shown to relieve suppression of BCL6 target genes and inhibit growth of lymphoma cells *in vitro*. Tumor growth inhibition in mouse DLBCL xenograft models has been reported for several BCL6 inhibitors. However, their use is limited due to the low binding affinity of most of these molecules [22–24]. Despite recent advances in developing BCL6 inhibitors [19, 25–28], no compound has yet reached the clinic. Furthermore, there exist controversies around the rationale and the impact of targeting BCL6 as a monotherapy due to the presence of high intra- and inter-tumor heterogeneity regarding type and number of oncogenic mutations [2, 3] and the possibility of oncogene addiction switching following BCL6 targeted therapies by reactivating BCL2-family dependent anti-apoptotic pathways [29].

We have recently reported highly selective BCL6 inhibitors and degraders with nanomolar potency *in vitro* [30]. Importantly, we found that the anti-proliferative activity of BCL6 degraders such as BI-3802 on tissue culture cells is generally higher than that of BCL6 inhibitors despite their equipotent BCL6 binding affinities. Therefore, BCL6 degradation is considered as a promising and novel strategy for BCL6-targeted therapies. Pharmacokinetic properties, however, limit the use of these BCL6-degrading compounds *in vivo*, such that the effect of BCL6 degradation on *in vivo* growth of lymphoma cells cannot be studied. Addressing this question, we report on the establishment of an inducible BCL6 knock-out DLBCL model, which allows studying the phenotype of BCL6 loss in DLBCL xenografts *in vivo*.

## RESULTS

### Negative effects of BCL6 knock-out on DLBCL cell growth

**Figure 1:**
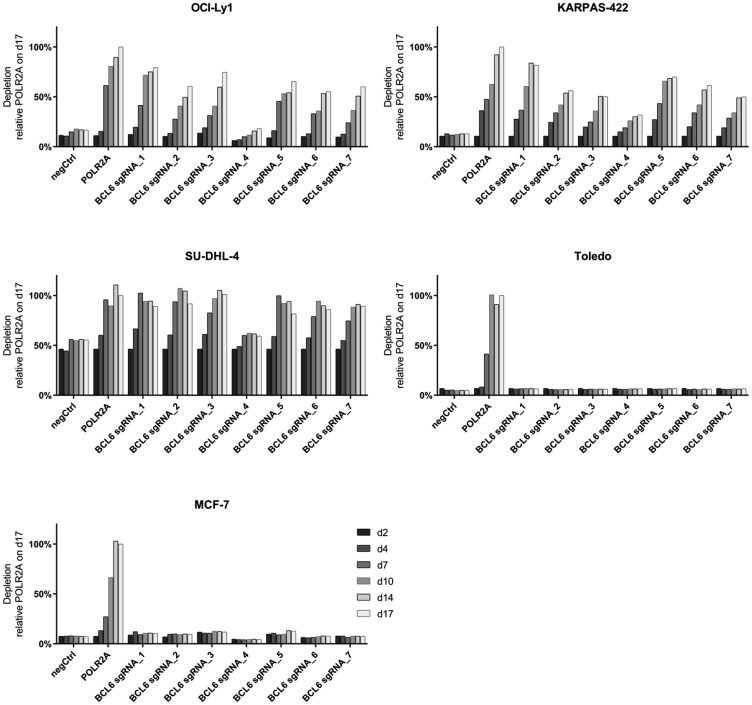
Depletion of BCL6 knock-out DLBCL cells in bulk assays. A time course CRISPR depletion experiment, following the depletion kinetics of GFP^+^ cells (Cas9 and sgRNA expressing) relative to the GFP^-^ cells (Cas9-expressing) in the DLBCL cell lines OCI-Ly1, KARPAS-422, SU-DHL-4 and Toledo and the breast cancer cell line MCF-7. POLR2A serves as a core essential control gene. NegCtrl depicts a non-targeting control and BCL6 sgRNAs 1–7 are BCL6 specific sgRNAs. Data are shown as relative GFP expression to the pos Ctrl sgRNA POLR2A on day 17 post infection.

We performed gene knock-out studies using the CRISPR/Cas9 system to address the dependency of different DLBCL cell lines on BCL6 (Figure 1). OCI-Ly1, KARPAS-422 and SU-DHL-4 cells stably expressing Cas9 were infected with sgRNAs targeting BCL6 at 7 different genomic sites (1–2 in the BTB domain; 3–7 in zinc finger domains). The effect of each sgRNA on cell survival was determined by monitoring the proportion of GFP^+^ cells (sgRNA expressing) vs. GFP^–^ cells in a bulk depletion assay. We observed that an RNA polymerase II subunit A (POLR2A) targeting sgRNA, which was used as a positive control, caused a rapid depletion of transduced cells within 4–7 days post infection. Targeting BCL6 with different sgRNAs showed a comparable kinetic and magnitude of effect. The DLBCL cell line Toledo and the breast cancer cell line MCF-7, which both lack expression of BCL6, were used as controls and did not show depletion following infection with BCL6 targeting sgRNAs. These results indicated that BCL6 is an essential gene in BCL6 expressing DLBCL cell lines.

### Establishment of an inducible CRISPR/Cas9 system to conditionally knock-out BCL6 in DLBCL

To further explore cellular and molecular functions of BCL6 in DLBCL and investigate its role in tumor maintenance *in vivo*, we devised a Doxycycline (DOX)-inducible CRISPR/Cas9 approach that enables conditional BCL6 knock-out in established DLBCL tumors (Supplementary Figure 1). To this end, we sequentially transduced SU-DHL-4 cells with lentiviral vectors expressing the reverse Tet transactivator (rtTA3) and a DOX-inducible Cas9: P2A: GFP transgene under control of an improved Tet-responsive element promoter (TRE3G; Supplementary Figure 1A). Single cell derived clones displaying high levels of Cas9: P2A: GFP induction upon DOX treatment were tested for tightly controllable and efficient CRISPR/Cas9 editing using an sgRNA targeting the surface molecule CD46. Clones were deemed non-leaky if sgRNA transduced cells did not show changes in CD46 surface expression over prolonged culture periods (up to 21 days) in the absence of DOX (Supplementary Figure 1B).

An appropriate SU-DHL-4 Cas9 clone was then transduced with a lentiviral vector co-expressing mCherry and an sgRNA targeting the BTB domain of BCL6 or a negative control sgRNA (Supplementary Figure 1C). The editing efficiency was confirmed in bulk depletion assays after DOX induction of Cas9 (Supplementary Figure 1C, left panel). In BCL6 sgRNA infected cells, DOX-induction led to efficient depletion of mCherry^+^ cells (reflecting BCL6 knock-out cells) with less than 10% mCherry^+^ cells remaining after 10 days of DOX treatment. In contrast, the proportion of mCherry^+^ cells in negative control sgRNA infected cells remained unaffected during 22 days of DOX treatment. DOX titrations from 1–500 ng/ml revealed that a concentration of 100 ng/ml was sufficient to induce maximal GFP expression after 48 h and this concentration was therefore chosen for further experiments (Supplementary Figure 2).

**Figure 2:**
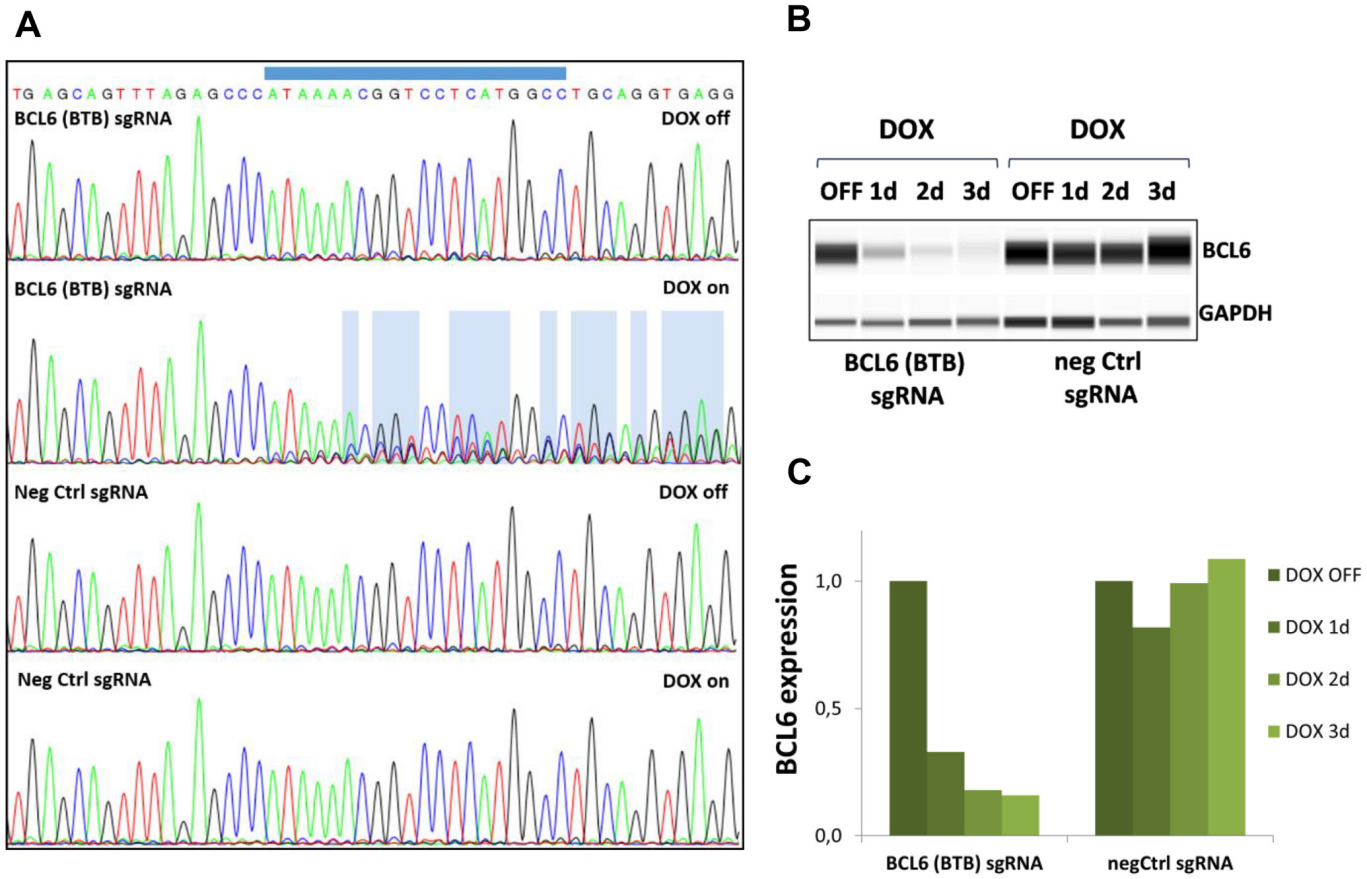
Gene editing and BCL6 protein deletion in SU-DHL-4 Cas9 cells. (**A**) Sanger sequencing reads obtained from SU-DHL-4 cells expressing negative control sgRNA or BCL6 (BTB) targeting sgRNA after 5 days vehicle (DOX off) or DOX (DOX on) treatment. The BCL6 sgRNA binding site is indicated on top. DNA sequence changes are highlighted in blue. (**B**) The loss of BCL6 protein after DOX treatment at the indicated time points was investigated using WES protein analysis using GAPDH as a loading control. One representative blot is shown for each cell line. (**C**) Quantification of BCL6 protein levels normalized to GAPDH and relative to DOX off. Data shown depict the mean of two biological replicates.

We then validated that the Cas9 protein levels expressed from the inducible vector led to efficient loss of BCL6 protein. To address this, sgRNA containing mCherry^+^ cells were purified (as indicated in Supplementary Figure 1C, right panel) and BCL6 gene editing at the sgRNA target locus and deletion of BCL6 protein after DOX treatment were evaluated (Figure 2). DOX-induced gene editing revealed changes in sequence reads in BCL6 sgRNA infected SU-DHL-4 after DOX induction (DOX on) but not in uninduced (DOX off) or negative control sgRNA infected cells (DOX off or on) (Figure 2A). Reduced BCL6 protein levels in BCL6 sgRNA-infected cells were observed one day after DOX treatment and after three days, BCL6 protein was below the detection limit while remaining unaltered in negative control cells (Figure 2B, 2C). Collectively, these data demonstrate that the inducible CRISPR/Cas9 system leads to efficient BCL6 knock-out and can be used to investigate the cellular effects in response to genetic loss of BCL6.

### Conditional BCL6 knock-out in SU-DHL-4 cells *in vitro* induces growth arrest

**Figure 3:**
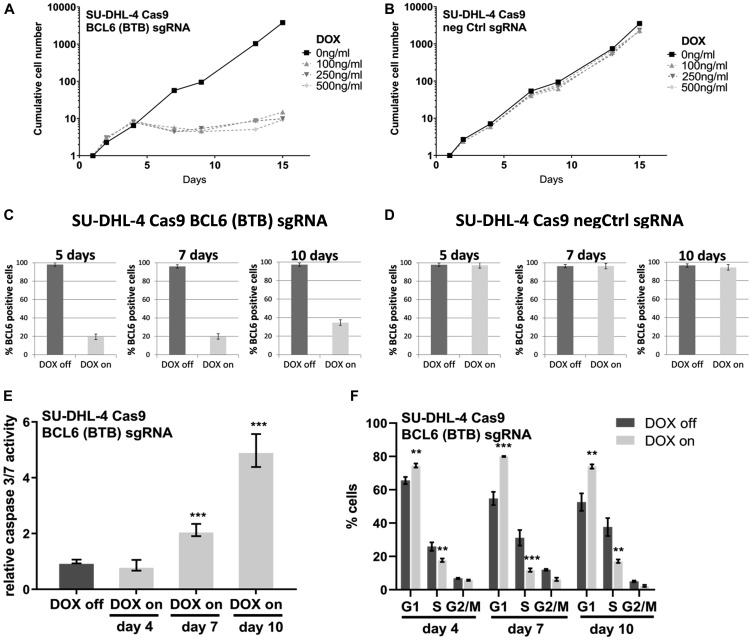
Conditional BCL6 knock-out in SU-DHL-4 *in vitro* induces anti-proliferative effects. Long-term proliferation assays with (**A**) BCL6 sgRNA and (**B**) negative control infected SU-DHL-4 Cas9 cells after DOX induction. For this assay cells were kept at constant concentrations of 3 different DOX concentrations as indicated and split to 200,000 cells per ml every 3–4 days. Split rates were multiplied to derive growth curves. BCL6 protein expression was determined at the indicated time points after DOX induction (100 ng/ml) in (**C**) BCL6 and (**D**) control sgRNA SU-DHL-4 Cas9 cells after immunohistochemical staining of cell pellets. (**E**) Caspase 3/7 activity and (**F**) cell cycle analysis after 4–10 days DOX treatment were investigated in SU-DHL-4 BCL6 sgRNA transduced cells. Data are shown as means ± SD of independent experiments (*n* = 2 – 8). ^**^ *p* ≤ 0.01; ^***^ *p* ≤ 0.001.

We next determined whether conditional loss of BCL6 affects lymphoma cell proliferation and/or survival *in vitro* (Figure 3). Induction of Cas9 caused an arrest in proliferation after 4–7 days in SU-DHL-4 cells expressing BCL6 targeting sgRNA (Figure 3A) but not in negative control cells (Figure 3B). Quantification of the proportion of BCL6-expressing cells after 5 and 7 days of DOX treatment revealed the presence of 20% BCL6 positive cells (Figure 3C). After 10 days, the percentage of BCL6-expressing cells rose to 35%, indicating a growth advantage for those cells. In contrast, DOX treatment in control cells did not have any effects on BCL6 expression (Figure 3D). With the deletion of BCL6, a significant induction of Caspase 3/7 activity was detectable after 7 and 10 days, indicating that apoptosis plays a major role in the curbed proliferation (Figure 3E). Furthermore, DOX treatment caused a significant elevation of SU-DHL-4 cells in the G1-phase of the cell cycle at all investigated time points (Figure 3F). These results suggest that genetic BCL6 loss inhibits cell proliferation by inducing a cell cycle arrest together with significant effects on apoptosis in the SU-DHL-4 lymphoma cell line.

### Comparable effects after BCL6 knock-out and compound induced BCL6 degradation

**Figure 4:**
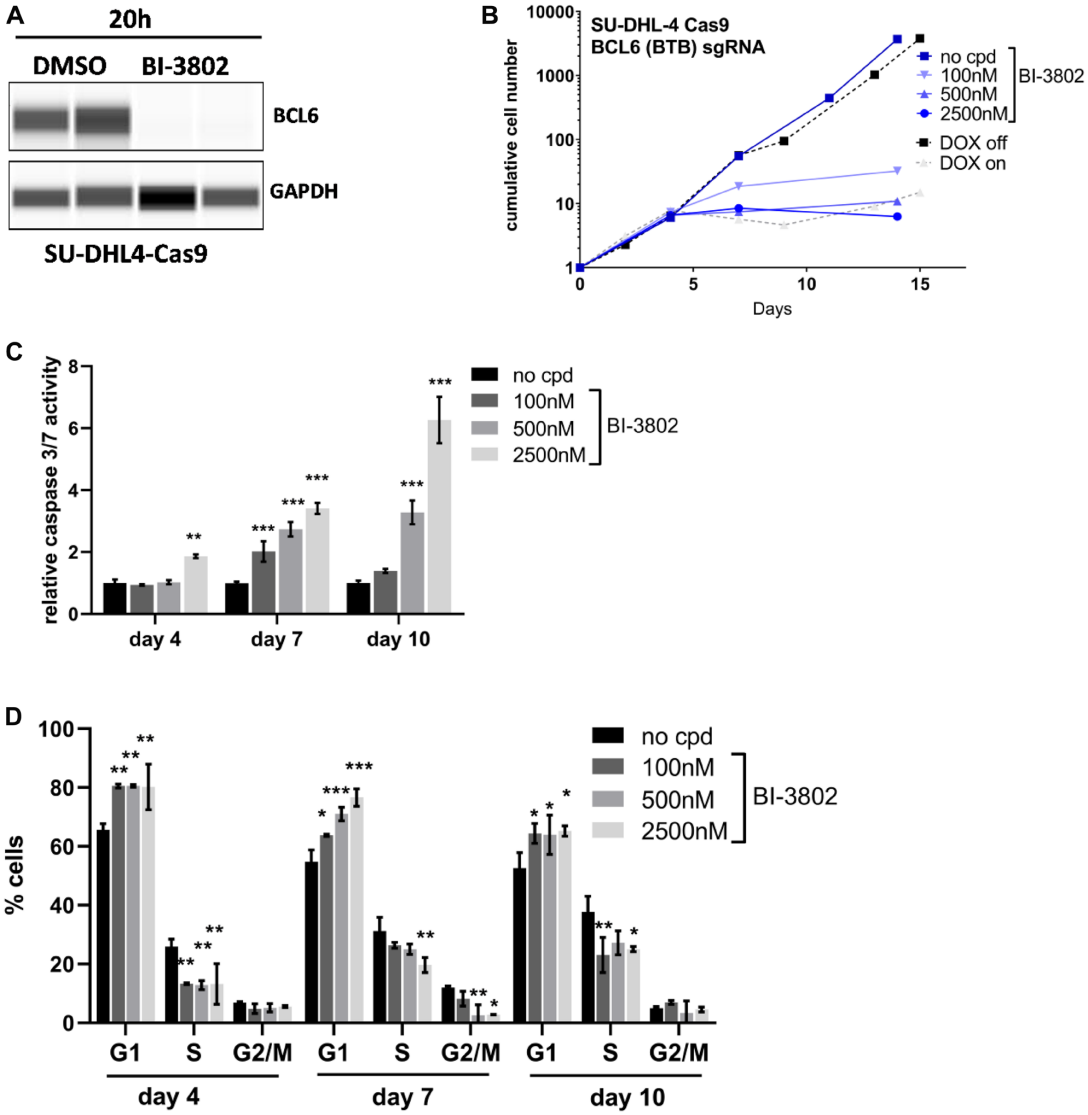
Comparable effects after BCL6 degradation and knock-out. (**A**) WES protein analysis of BCL6 in SU-DHL-4 Cas9 cells after BI-3802 treatment (500 nM, 20 h). (**B**) Long-term proliferation assays with BCL6 sgRNA infected SU-DHL-4 Cas9 cells after DOX induction and BCL6 degrader treatment. (**C**) Caspase 3/7 activity and (**D**) cell cycle analysis after 4–10 days treatment with BI-3802 at the indicated concentrations. Data are shown as means ± SD of independent experiments (*n* = 2 – 6). ^*^ *p* < 0.05; ^**^ *p* ≤ 0.01; ^***^ *p* ≤ 0.001.

In a recent publication we showed that BCL6 protein degradation using the small molecule BCL6 degrader BI-3802 curbs proliferation in various DLBCL cell lines *in vitro* [30]. Also in the SU-DHL-4 Cas9 clone BCL6 protein degradation could be observed after 20 h treatment with BI-3802 at 500 nM (Figure 4A). In order to compare the pharmacologically induced loss of BCL6 protein to genetic loss of BCL6, we treated the inducible SU-DHL-4 cell line with DOX or the BCL6 degrader BI-3802 at different concentrations (100 nM, 500 nM, and 2500 nM). BI-3802 treatment showed an anti-proliferative effect in a dose- and time-dependent manner (Figure 4B). At concentrations of 500 nM and 2500 nM, BI-3802 had comparable effects on proliferation as induced by knock-out of BCL6. These observations could be confirmed in another DLBCL cell line, KARPAS-422, where 2 independent clones were characterized (Supplementary Figure 3). When determining apoptosis after 4, 7, and 10 days of treatment we observed a significant induction of apoptosis at various concentrations of BI-3802 (Figure 4C). Cell cycle analysis revealed that BI-3802 resulted in a modulation of the cell cycle in a concentration dependent manner with a significantly increased proportion of cells in the G1-phase after 4, 7 and 10 days of treatment (Figure 4D). Taken together, these results indicate comparable effects on proliferation, apoptosis, and cell cycle after genetic and BI-3802 degrader-induced BCL6 loss in SU-DHL-4 cells.

**Figure 5:**
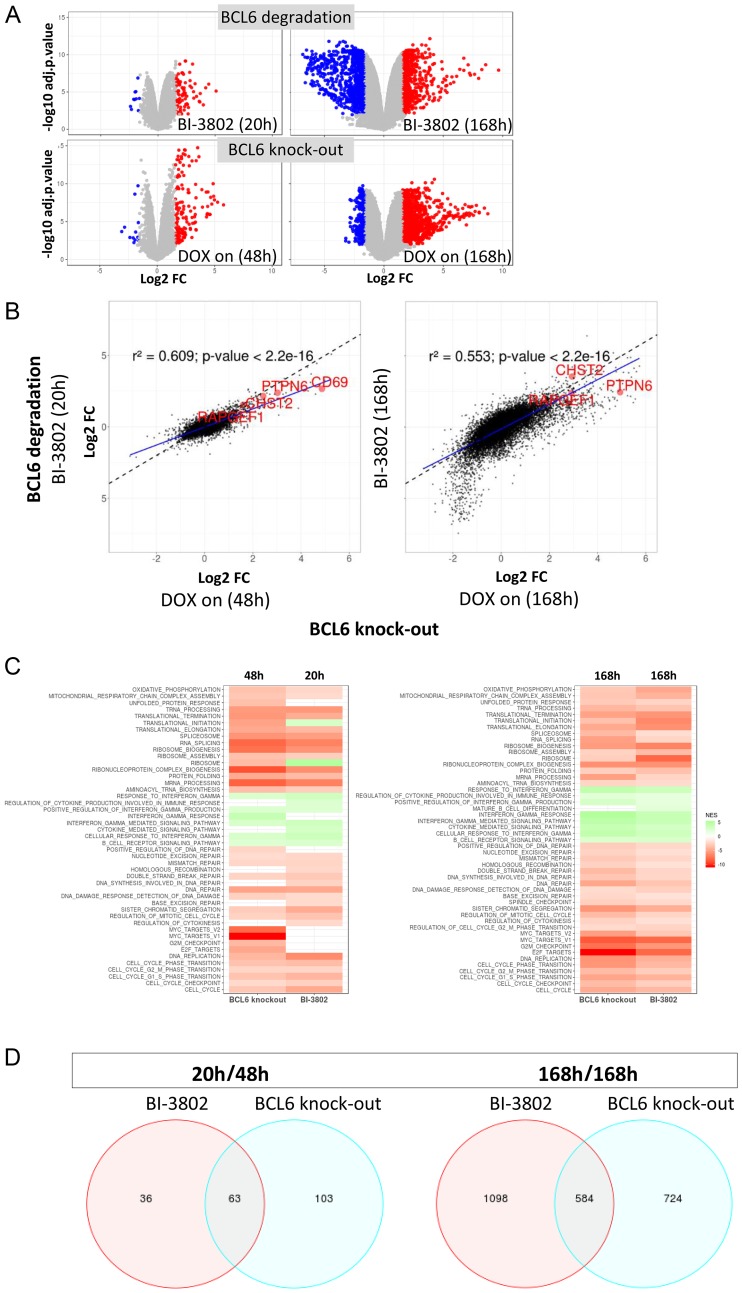
Conditional BCL6 knock-out in SU-DHL-4 induces gene perturbations similar to BCL6 degradation. RNA-seq analysis was performed to compare gene expression after BCL6 knock-out and compound-induced degradation. (**A**) Volcano plot visualizing log2-scaled fold changes (x-axis) induced by either BI-3802 mediated degradation (compared to DMSO treatment) or BCL6 knock-out (compared to control sgRNA treatment) versus statistical significances (-log10 of the adj. *p*-value on the y-axis). Significantly deregulated genes (adj. *p*-value ≤ 0.01, fold change ≥ 3) are depicted in blue and red for repressed and induced genes, respectively. (**B**) Correlation of changes in gene expression induced by BCL6 knock-out (x-axis) or BI-3802 mediated degradation (y-axis). Genes near the dotted lines show comparable expression modulation in the BI-3802 treated data versus the BCL6 knock-out data set. Blue lines show linear regressions of the actual fold-change values. The goodness-of-fit of the linear regressions are shown by the r^2^ value in the graphs. (**C**) Gene set enrichment analysis (selected terms, FDR ≤ 0.1) reflecting genes set that are enriched/depleted for genes modulated by BCL6 knock-out or BI-3802 mediated degradation. The normalized enrichment score (NES) is color-coded in the heatmap. Negative values indicate gene sets that are significantly enriched for genes that are down-regulated upon BCL6 knock-out or BI-3802 treatment as shown in Supplementary Figure 4C (cell cycle). (**D**) Venn diagram indicating the overlap of genes after BCL6 degradation and BCL6 knock-out in SU-DHL-4 cells at the indicated time points of BI-3802 and DOX treatment.

Next, we were interested in testing if the effects of BCL6 knock-out are comparable to BCL6 degradation at a molecular level, i. e. if the same set of genes is altered in both cases. To test this we performed genome-wide gene expression studies using RNA-seq with the inducible SU-DHL-4 Cas9 clones (negCtrl and BCL6 sgRNA). This analysis was performed in triplicates. RNA from cells was harvested after 48 h and 168 h of DOX treatment and the transcriptional profile was compared to SU-DHL-4 cells in the presence of BI-3802, as published earlier [30]. At early time points (degradation: 20 h, knock-out: 48 h) both treatments resulted in more up- than down-regulated genes, the knock-out showing a stronger effect (154 vs. 89 genes; Figure 5A, Supplementary Table 1, Supplementary Table 2). At the later time point (168 h), BCL6 knock-out resulted in a stronger increase of up-regulated genes than the BCL6 degrader BI-3802 (1037 vs. 656), while BCL6 degradation led to more complex down-regulation effects (1026 vs. 271). Gene ontology analysis revealed that these downregulated genes after BI-3802 treatment were predominantly associated with cell cycle control (Supplementary Figure 4B). When compared across both treatment conditions, there was a significant correlation (*p*-value < 2.2e-16) on the changes in gene levels induced upon BCL6 knock-out with the effects of BI-3802. Genes induced by BCL6 knock-out and degradation include several known BCL6-regulated genes, such as CHST2, PTPN6, RAPGEF1 and CD69, which are highlighted in Figure 5B. Perhaps not surprisingly, the magnitude of transcriptional changes was more pronounced after BCL6 knock-out as visualized by the regression line lying below the diagonal (y = x) line (Figure 5B). Gene set enrichment analysis showed down-regulation of cell cycle, DNA repair and protein synthesis related pathways upon loss of BCL6, both via genetic and pharmacological approaches (Figure 5C, Supplementary Figure 4C). Further pathway analysis of differentially regulated genes revealed immune-response pathways like interferon-γ or B-cell receptor signaling to be upregulated (Supplementary Figure 4C; Supplementary Table 3). A common set of 63 genes was found to intersect in BI-3802 treated and BCL6 knock-out cells after 20 h and 48 h, respectively, while after 168 h 584 genes were commonly regulated by BCL6 degradation and BCL6 knock-out (Figure 5D, Supplementary Table 2). Taken together, the effects of BCL6 knock-out and compound-induced degradation on gene expression are highly correlated and show a similar profile of pathway modulation, confirming that they can be attributed to the specific loss of BCL6 in both cases. These results together highlight the value of the BCL6 degrader BI-3802 in selectively and potently inhibiting BCL6 function.

### BCL6 knock-out in a DLBCL xenograft induces tumor stasis

**Figure 6:**
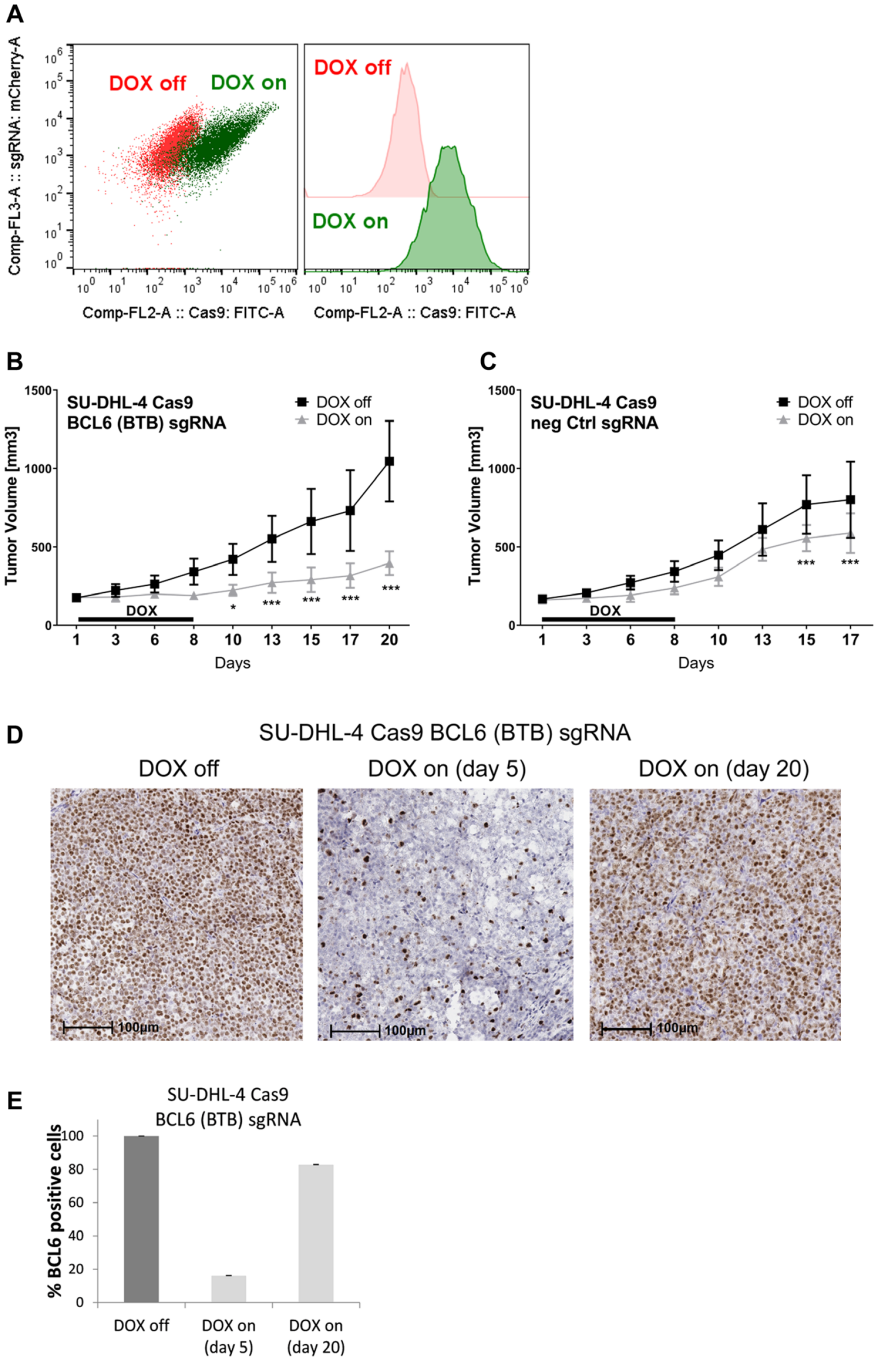
BCL6 knock-out in a DLBCL xenograft induces tumor stasis. Tumor xenografts were established in C.B-17 SCID mice by subcutaneous injection of inducible SU-DHL-4 Cas9 BCL6 and control sgRNA cells. Mice were randomized to receive drinking water with DOX (2 mg/kg) plus 5% sucrose (DOX on) or 5% sucrose only (DOX off). (**A**) After 5 days DOX treatment tumors from four mice were harvested and analyzed for Cas9 GFP induction using flow cytometry. Cas9-GFP-induced cells are indicated in green, non-induced cells in red. (**B–E**) Tumor-bearing mice were treated with DOX for 8 days after which tumors from control and BCL6 knock-out tumors were harvested 17/20 days after start of DOX treatment, respectively. Tumor volumes from (B) BCL6 sgRNA tumors (*n* = 10 DOX off, *n* = 7 DOX on) and (C) control (*n* = 10 DOX off, *n* = 8 DOX on) were measured. ^*^ *p* < 0.05; ^***^ *p* ≤ 0.001. (D) Tumor BCL6 protein levels were determined using IHC analysis. Representative images of BCL6 IHC staining in SU-DHL-4 tumors are shown. Scale bars 100 μm. (E) Quantification of BCL6 positive cells in SU-DHL-4 BCL6 sgRNA tumor sections after vehicle (DOX off) and DOX treatment (5 days and 20 days after start of DOX treatment). Data are shown as means ± SD relative to DOX off (*n* = 4 – 10).

Since the poor bioavailability of BI-3802 does not permit its use in animals, we wanted to apply the inducible knock-out system to investigate the effects of BCL6 depletion on tumor growth *in vivo*. Therefore we first examined the engraftment and growth properties of the inducible SU-DHL-4 Cas9 cells *in vivo*. SU-DHL-4 control cells and BCL6 sgRNA cells showed a comparable tumor growth (data not shown). To assess the effect of BCL6 loss on tumor growth, Cas9 expression was induced (DOX on for 8 days) in animals (*n* = 10) harboring established tumors (150–250 mm^3^) and tumor growth was monitored. Effective induction of GFP expression *in vivo* (reflecting Cas9 induction) was determined in tumors 5 days after DOX treatment (Figure 6A). In control xenograft tumors, DOX treatment had a minor effect on tumor growth, resulting in a significantly reduced tumor volume 15 and 17 days after start of DOX treatment (Figure 6C). In BCL6 sgRNA tumors, DOX treatment led to tumor stasis 6 days after treatment, with a maximal tumor growth inhibition of 73% achieved after 20 days (Figure 6B). The initial tumor stasis in BCL6 knock-out tumors was followed by a slow but continuous tumor growth beginning around day 13 of the treatment. Tumors from the remaining mice were harvested 20 days after start of DOX treatment and BCL6 protein levels were determined (Figure 6D). Immunohistochemical (IHC) analysis revealed that at this time-point the fraction of BCL6 expressing cells in the DOX treated mice was 82%, significantly higher than after 5 days of treatment (Figure 6E), indicating that the positive selection of BCL6 expressing cells seen *in vitro* (Figure 3C) also occurs *in vivo*. Collectively, xenograft studies demonstrate that the inducible knock-out DLBCL cell line works highly efficiently also *in vivo*. Targeting BCL6 in a DLBCL xenograft controlled tumor growth *in vivo*, which was characterized by a significant tumor growth inhibition. Initial tumor stasis was followed by slow tumor growth, which can be attributed to the selection of cells lacking a functional BCL6 knock-out. In summary, this indicates that targeting of BCL6 represents a viable strategy for lymphoma treatment.

### Data availability

RNA sequencing data are deposited at NCBI Gene Expression Omnibus (accession number GSE127266).

## DISCUSSION

DLBCL is a complex, multi-hit disease of B-cells with a diverse range of aberrant oncogenic signaling pathways [31]. Targeting specific oncogene dependencies within the DLBCL subgroups offers a more precise approach to treat cancer patients compared to standard chemotherapy-based approaches. Effective targeted therapies require the identification of essential oncogenic pathways within distinct DLBCL subgroups [4]. Genetic studies using BCL6 shRNA in DLBCL cell lines have demonstrated a requirement of BCL6 for viability and proliferation *in vitro* [22]. Furthermore, BCL6-dependency of lymphoma cell lines, including SU-DHL-4, was observed in functional CRISPR screens with BCL6 being among the most significant hits [3]. To date the evaluation of the effects of targeting BCL6 *in vitro* and *in vivo* has been limited to the use of low affinity binding BCL6 inhibitors at high concentrations [22–24, 32]. Recently, McCoull et al. have developed highly potent inhibitors of BCL6, which, however, did not show significant anti-proliferative effects on lymphoma cells [28]. Further approaches to use BCL6 small molecule inhibitors as target binding ligand of proteolysis targeting chimeras (PROTACs) resulted in compounds that induce degradation of BCL6, albeit not to complete levels. However, also these PROTACs failed to induce enhanced anti-proliferative effects *in vitro* [19].

Inducible knock-out models are important tools to investigate whether a specific gene is essential for cell survival. Previous efforts to employ a DOX-inducible Cas9 approach for the conditional deletion of MCL-1 were hampered by inefficient induction of Cas9 expression [33]. In recent studies, robust expression of Cas9 in an *in vivo* Cas9 expressing mouse model was reported [34, 35]. Here, we have demonstrated that our system permits effective, conditional expression of Cas9 in DLBCL cell lines. Importantly, our system revealed no premature Cas9 expression due to promoter leakiness prior to DOX induction, which is important to limit unregulated genome editing.

An anti-proliferative response was observed 4–7 days after deletion of BCL6 *in vitro* whereas tumor stasis occurred in *in vivo* xenograft studies. The genetic loss of BCL6 resulted in an inhibition of cancer cell proliferation and an arrest in cell cycle progression at the G1 transition with significant induction of apoptosis. This observation is supported by a downregulation of cell cycle-associated genes. CRISPR/Cas9 genome editing frequently generates in-frame mutations instead of insertions or deletions, and thus a certain percentage of cells with silent or non-functional mutations are expected [36]. Also in our model the editing efficiency of CRISPR on BCL6 is not 100% as a low percentage of tumor cells maintain BCL6 expression after DOX treatment (Figure 3C). This subpopulation of BCL6 expressing cells increases over time, both *in vitro* and *in vivo* and contribute to a continuous tumor growth. At the time of tumor stasis (up to 8 days after start of DOX treatment) the xenografts contain 15% tumor cells which still express BCL6. This eventual expansion of the tumor escaper cells limits the time window in which the effects of BCL6 knock-out can be observed. Thus, it is well possible that the effects of continuous and effective inhibition of BCL6 exceed the tumor stasis observed in our model. Further, it is important to mention that DLBCL cell lines are among the most robustly and aggressively growing lymphomas, since most of the explanted lymphoma cells do not replicate and survive for longer periods *ex vivo*. A concurrent high expression of MYC and BCL2 [37] and mutant p53 [38] has been linked to unfavorable treatment responses and poor prognosis in DLBCL patients. Indeed, the SU-DHL-4 DLBCL cell line, which expresses wild-type MYC and mutant BCL2 and p53, only display temporary responses to the standard of care treatment R-CHOP, but shows no regressions in xenograft experiments [39].

The finding of a comparable growth and transcriptional response after treatment with the BCL6 degrading compound BI-3802 and BCL6 knock-out *in vitro* suggests BCL6 degradation as an effective and promising therapeutic approach. Further optimization of small molecule degraders is needed to provide bioavailable compounds with high BCL6 binding potency, which allows pre-clinical studies in lymphoma models *in vivo*.

In summary, our findings have important implications for understanding the impact of BCL6-targeted therapies in DLBCL. According to our studies it is reasonable to predict that treatment of DLBCL with BCL6 degraders results in significant tumor growth inhibition and at least tumor stasis. The observed magnitude of effects of BCL6 blockade in monotherapy might provide a rationale for therapeutic combinations with other targeted and/or chemotherapeutic agents. Our CRISPR/Cas9 BCL6 knock-out model represents a valuable pre-clinical tool to evaluate such combination approaches.

## MATERIALS AND METHODS

### Cell culture

The tumor cell lines SU-DHL-4, KARPAS-422, OCI-Ly1, Toledo, MCF-7, and HEK293T were obtained from the American Type Culture Collection (ATCC) or the German Collection of Microorganisms and Cell Culture (DSMZ). All cell lines used in this study were cultured according to the manufacturer’s instructions.

### Lentiviral transduction of cell lines

Lentiviral particles were produced by transient transfection of HEK293T cells grown in 10-cm petri dishes with 15 µg of vector DNA along with the packaging constructs pcDNA3. GP.4xCTE gagpol (7 µg), pMD. G VSVG (1 µg), and pRSV-rev (5 µg) using standard calcium phosphate precipitation (Invitrogen #K278001). Virus-containing supernatants were collected 48–72 h after transfection and passed through a 0.45 µm filter.

Constitutively Cas9 expressing cell lines, which were generated after lentiviral transduction and using puromycin as selection marker, were further transduced with sgRNA-encoding vectors harboring a GFP fluorescence marker. On day 3 post infection a bulk depletion assay was performed, in which the percent GFP expression was recorded at the indicated time points by flow cytometry (Accuri C6, BD Biosciences). GFP expression was then normalized to the pos Ctrl sgRNA POLR2A on day 17 post infection. Non-targeting sgRNAs were used as negative control sgRNAs.

For the generation of inducible cell lines, SU-DHL-4 or KARPAS-422 cells were transduced with a lentivirus co-expressing the reverse tetracycline-controlled transactivator 3 (rtTA3), the ecotropic receptor (EcoR) and a puromycin selection cassette (pLenti-EF1a-rtTA3-IRES-EcoR-PGK-Puro) and selected with puromycin. Selected cells were then transduced with a lentivirus expressing spCas9 and GFP from an improved tetracycline-responsive element promoter (pLenti-TRE3G-Cas9-P2A-GFP). Following Cas9 induction using doxycycline (DOX) treatment (Sigma #D9891) (1 µg/ml) for 48 h, Cas9/GFP expressing single cell clones were isolated using FACS sorting (Sony Sorter SH800), expanded and tested for promoter leakiness as follows: Individual SU-DHL-4-Cas9 clones were infected with sgRNA expressing construct targeting the surface molecule CD46. Transduced cells were cultured up to 21 days during which the expression of surface CD46 was monitored using FACS staining (Biolegend #352408) and compared to negative control sgRNA infected cells.

A selected SU-DHL-4 Cas9 or KARPAS-422 Cas9 clone was infected with a lentiviral vector co-expressing sgRNAs and an improved tracr scaffold [40] from a human U6 promoter and the mCherry fluorescent protein from a minimal EF1a promoter (pLenti-U6-sgRNA. iT-EF1a-mCherry). Cas9-editing efficiency was confirmed in a bulk depletion assay after DOX addition. The percentage of mCherry expressing cells was recorded at the indicated time points by flow cytometry (Accuri C6, BD Biosciences) and compared to negative control sgRNA infected cells. sgRNA/mCherry expressing single cell clones were FACS sorted and selected clones were used for further experiments.

The following sgRNA sequences were used:

BCL6 sgRNA_1: 5′-GGCCATGAGGACCGTTTTAT-3′.

BCL6 sgRNA_2: 5′-ATCTCGGCTCAATTTGCGGG-3′.

BCL6 sgRNA_3: 5′-CTGAGGAGGCCTCACTCAAG-3′.

BCL6 sgRNA_4: 5′-GAGGTTGCCCTTGTAGCGGA-3′.

BCL6 sgRNA_5: 5′-GGTTGGCTGGCCGGTTGAAC-3′.

BCL6 sgRNA_6: 5′-CTGTACAAATCTGGCTCCGC-3′.

BCL6 sgRNA_7: 5′-AAATCTGTGGCACCCGTTTC-3′.

negCtrl sgRNA: 5′-GATACACGAAGCATCACTAG-3′.

POLR2A sgRNA: 5′-GTACAATGCAGACTTTGACG-3′.

CD46 sgRNA: 5′-GGATCAGTAGCAATTTGGAG-3′.

### Sanger sequencing of sgRNA target site

Genomic DNA was isolated from cells using the QIAamp DNA Mini Kit (Qiagen #51304). Cloning of the target site and DNA sequencing was performed by Eurofins (Ebersberg, Germany). Primers were designed to span the expected indel positions in the genomic DNA (BCL6-1F1 5′ - GAAGAATAATGGCCAGAGTTGGAC-3, BCL6-1R1 5′ - TGGCTCTTTCTTTTCTAAAAGTGCATTC-3). The PCR cycling conditions were as follows (PCR 1: 95°C 2 min [95°C 1 min, 57°C 30 s, 72°C 1 min] x 35, 72°C 10 min, 4°C hold step). PCR amplification was performed using GoTaq HotStart Green MasterMix (Promega). For PCR reactions peqStar 96 HPL (PEQLAB Biotechnologie) and/or GeneTouch (Biozym Scientific) and/or Biometra Tadvanced (Biometra) thermal cyclers were used. The amplicon size generated was 542 bp. Successful and specific PCR amplification was verified by agarose gel electrophoresis. PCR products were purified by performing a precipitation step applying polyethyleneglycol (PEG). PCR product quantity was estimated using agarose gel electrophoresis by visual comparison to a reference standard. Approximately 5–10 ng of the PCR product were used as template per sequencing reaction.

All sequences were generated using BigDye terminator chemistry (version 3.1), if necessary in combination with dGTP BigDye terminator chemistry (version 3.0) (Thermo Fisher Scientific). Sequencing reaction cleanup was done either manually or on a Hamilton Starlet robotic workstation (Hamilton Robotics) by gel-filtration through a hydrated Sephadex matrix filled into appropriate 96-well filter plates followed by a subsequent centrifugation step. Finally all reactions were run on ABI3730xl capillary sequencers equipped with 50 cm capillaries and POP7 polymer (Thermo Fisher Scientific). Sequencing data was called using the original Sequencing Analysis Software 6 (Applied Biosystems) including the KB-basecaller (Thermo Fisher Scientific), which assigns quality values to all called bases similar to PHRED quality score [41]. Additional basecalling was performed using the PeakTrace basecaller from Nucleics Pty Ltd (Woollahra, AUS) to improve the single peak resolution and quality values and therefore increase the reading lengths. The assembly was performed using the Staden Software Package (Roger Staden, LMB/ Pregap4 version 1.6, Gap4 version 4.11.2). Visualization of the sequencing reads was performed with the R package ‘sangerseqR’ (http://www.bioconductor.org).

### Drug treatments and functional assays

For long-term proliferation assays, cells were inoculated at a density of 200,000 cells per ml in 1.5 ml in 24-well plates. DOX, compound (BI-3802) or DMSO were added, and cells were split to 200,000 cells per ml every 3 to 4 days. Upon splitting, fresh compound/DOX was added to keep the concentration constant. Split rates were multiplied to derive proliferation factors.

For cell cycle analysis, cells (5 × 10^6^) were collected and fixed in 2.5 ml of cold Cytofix/Cytoperm (BD Biosciences #554722) for 20 min at 4°C. After centrifugation at 400 × g for 5 min the cell pellets were washed twice in 10 ml Perm/Wash buffer (BD Biosciences #554723), and then centrifuged again at 400 × g for 5 min. The cell pellets were stained with 0.5 ml Perm/Wash buffer containing 1 μg/ml DAPI (BD Biosciences #564907) at RT for 15 min. Cells (2 × 10^4^) were analyzed by flow cytometry (FACS Canto II, BD Biosciences) and the analysis was performed using FlowJo Software with Dean-Jett-Fox cell cycle modeling.

The Caspase-Glo 3/7 assay reagent (Promega #G8093) was used for measuring apoptosis in DOX-induced cells *in vitro*. For this, cells were seeded at a density of 3,000 cells in a 96-well plate and after 4, 7, and 10 days the Caspase-Glo 3/7 reagent was added directly to the cells. After 60 min incubation at RT luminescence was determined using an EnSpire Multimode Plate Reader 2300 (PerkinElmer). The amount of luminescence is proportional to the amount of caspase activity in the sample and was normalized to cell number determined using a PrestoBlue™ Cell Viability Reagent (Invitrogen #A13262).

### RNA isolation and preparation of sequencing libraries

For RNA-seq analysis negative control and BCL6 sgRNA infected SU-DHL-4 Cas9 cells were seeded at a density of 1 × 10^6^/ml and treated with DOX (100 ng/ml) for 48 h and 7 days. For 7 day treatments, cells were split once after 3 days and fresh DOX was added. All treatments were performed in triplicates. Total RNA was isolated using the RNeasy Plus Universal Mini kit (Qiagen, #73404). Instead of chloroform 10% volume 1-bromo-3-chloropropane (Sigma-Aldrich) was used. RNA sequencing libraries were prepared using the TruSeq RNA Library Preparation Kit v2 (Illumina) and subsequently sequenced on the Illumina NextSeq 500 system using a paired-end 76 bp protocol.

### Bioinformatics analysis

Sequencing reads from the RNA-seq experiment were processed with a pipeline building upon the implementation of the ENCODE’ “Long RNA-seq” pipeline: Filtered reads were mapped against the Homo sapiens (human) genome hg38/GRCh38 (primary assembly, excluding alternate contigs) using the STAR (v2.5.2b) [42] aligner allowing for soft clipping of adapter sequences. For quantification, transcript annotation files from Ensembl version 86 we used, which corresponds to GENCODE 25. Samples were quantified with the above annotations, using RSEM (v1.3.0) [43] and featureCount (v1.5.1) [44]. Quality controls were implemented using FastQC (v0.11.5) [45], picardmetrics (v0.2.4) (available online at: https://github.com/slowkow/picardmetrics), and dupRadar (v1.0.0) [46] at the respective steps. Two samples were excluded from the analysis due to poor sequencing quality (BCL6-sgRNA_Dox-on_2d_rep3_J22790, negCtrl-sgRNA_Dox-off_7d_rep2_J22792). PCA analysis (Supplementary Figure 4A) illustrates the variabilities in the individual samples. Two additional samples were excluded from the analysis due to their outlier behavior as shown in Supplementary Figure 4A (negCtrl-sgRNA_Dox-on_7d_rep1, BCL6-sgRNA_Dox-off_7d_rep2).

Differential expression analysis was performed on the mapped counts derived from featureCount using limma/voom [47, 48]. If not otherwise stated, an absolute log2 fold change cut-off of 1 and a false discovery rate (FDR) of ≤ 0.1 was used. Pathway analysis (GSEA Preranked, ranking: log2FoldChange, scoring scheme = ‘classical’, 1000 permutations), available online at: https://cloud.genepattern.org) was performed according to [49]. The following MSigDB gene sets were queried: hallmark gene sets, C2 sub-collection CP: Canonical pathways - KEGG, C5 collection: Gene Ontology (GO, biological processes), using standard settings, 1000 permutations (gene set) and a false discovery rate (FDR) of ≤ 0.1. GO term annotation was performed with clusterProfiler [50].

### Capillary Western blot (WES) analysis

Capillary western blot analysis was performed using the ProteinSimple WES System according to the manufacturer’s instructions. Cells (300,000) were collected by centrifugation, washed once with PBS, and lysed in 25 µl lysis buffer (1% Triton, 350 mM KCl, 10 mM Tris [pH 7.4]) supplemented with a phosphatase-protease inhibitor cocktail (Thermo Scientific, #1861281), 10 mM DTT, and Benzonase 0.5 µl/ml (Novagen #70746-10KU, 25 U/ml). Tumor homogenization was performed using a TissueLyser II (Qiagen) for 30 seconds at 30 Hz shaking with a 5 mm stainless steel bead (Qiagen, #69989), followed by a 30 min incubation time and centrifugation at 15000 × g for 10 min at 4°C. Supernatants were collected and protein concentrations were determined using a Bradford protein assay (BioRad #500-0006). BCL6 and GAPDH were identified with primary antibodies against BCL6 (Sigma #HPA004899, 1:50) and GAPDH (Abcam #9485, 1:1000), followed by immunodetection using Wes Master Kit HRP-conjugated anti-rabbit secondary antibody and chemiluminescent substrate (ProteinSimple #DM-001). Using Compass software, electropherograms were generated and the area under the curve was calculated. The area under the curve represents the signal intensity of the chemiluminescent reaction and is proportional to the amount of target protein in a respective capillary. BCL6 protein levels were normalized to GAPDH and are represented as relative to BCL6 levels in uninduced cells (DOX off) at the respective time points. Quantification data shown depict the mean of two biological replicates.

### Animal experiments

For subcutaneous xenograft models, 8 week old female C.B-17 SCID mice (C.B-Igh-1b/IcrTac-Prkdcscid, Taconic) were injected with 1 × 10^7^ SU-DHL-4 cells. Animals were randomized according to their tumor volumes when tumors reached diameters of approximately 150–250 mm^3^.

For induction of Cas9 expression *in vivo*, DOX was dissolved in sterile water and was administered in drinking water (2 mg/kg) plus 5% sucrose to cover the bitter taste. Drinking water containing DOX was replaced every 3 days due to the sensitivity of DOX to light. Mice were switched to drinking water supplemented with 5% sucrose plus DOX (2 mg/kg; DOX on; *n* = 10) or 5% sucrose only (DOX off; *n* = 10) for 8 days. Subcutaneous tumors were measured three times weekly using a caliper. Volumes were calculated according to the formula “tumor volume = length * diameter^2^ * π/6.” Tumor growth inhibition (TGI) was calculated to the formula: “TGI = 100 × (1-[(treated final day– treated day 1) / (control final day– control day 1)])”.

Animals were examined daily and euthanized based on severity criteria including body weight loss exceeding 18%. Of note, mice from both groups, either carrying control or BCL6 knock-out tumors displayed body weight reductions upon treatment with DOX –containing drinking water (Supplementary Figure 5) but recovered immediately after the treatment period of 8 days.

### Tumor dissociation and flow cytometric analysis

The tumors were dissected and tumor cells were isolated using gentleMACS dissociator (Miltenyi Biotech #130-096-730). In brief, the tumors were washed in PBS, cut into pieces using a scalpel and dispersed in dissociation mix. The tumor suspensions were transferred into gentleMACS C tubes (Miltenyi Biotech #130-096-334) shaken at 37°C and 100 rpm for 45 min using a gentleMACS dissociator. Cell suspensions were centrifuged at 400 × g and 4°C for 10 min, then re-suspended in PBS + 2% heat inactivated (hi) FCS. The tumor homogenates were filtered using a cell strainer (70 µm) and subsequently centrifuged at 400 × g and 4°C for 10 min. The cell pellets were incubated for 2 min on ice in 1 ml ACK lysis buffer (Gibco #A10492-01) and washed once with 10 ml PBS+2% hi FCS. The number of cells was determined using a Vi-Cell XR Cell viability analyzer (Beckmann Coulter) and GFP-expressing tumor cells were quantified after staining with the mouse CD45-BV421 (Biolegend, #30-F11) to exclude mouse immune cells and fixable viability dye eFl780 (eBioscience, #65-0865-14) to exclude dead cells. After 30 min of incubation at 4°C, cells were washed, resuspended in FACS stain buffer (BD Biosciences, # 554656), and analyzed (2 × 10^5^ gated on living cells).

### Immunohistochemical (IHC) staining of cell pellets and tumors

In a time course experiment, negative control and BCL6 sgRNAs infected SU-DHL-4 Cas9 cells were treated 5, 7, and 10 days with DOX (100 ng/ml) and paraffin-embedded cell pellets were prepared. Briefly, cells were washed with PBS, fixed for 10 min in 4% formalin, washed again, and then re-suspended in Histogel™ (Thermo Scientific). Cell pellets were embedded in paraffin using Histos 5 Rapid Microwave Histoprocessor (Milestone). Tumor samples were fixed in 4% formalin overnight and embedded in paraffin as described above. For IHC stainings paraffin blocks were sectioned (2 µm) and mounted on charged glass slides. Sections were dried and then de-paraffinated in 3 consecutive bathes of xylene, 100% EtOH, 96% EtOH, and 70% EtOH. For all stainings heat-induced epitope retrieval (HIER) was performed in an autoclave at low pH (Vector Laboratories). To avoid unspecific tissue peroxidase activity, the slides were incubated with 3% H_2_O_2_ for 5 min and then blocked with 5% goat serum in PBS. The primary antibody used was BCL6 (Cell Signaling #5650S, 1:50). After 1 h incubation, the staining was continued with three wash steps using PBS and secondary antibody (Dako EnVision) incubation for 30 min. Slides were then developed using 3,3′-diaminobenzidine (Sigma #D5905) dehydrated and counterstained with hematoxylin. Slides were scanned using the Aperio AT2 scanner (Leica Biosystems) and images analysis was performed using Tissue Studio 4.4.2 software (Definiens) for cell pellets and HALO digital image software 2.2 (Indica Labs) for tumor samples, respectively. The percentage of tumor cells staining positive for BCL6 was determined.

### Statistical analysis

Statistical analyses were performed using GraphPad Prism 8.0 software. Data were analyzed by ANOVA with the Bonferroni test for multiple comparisons. Data are expressed as mean ± SD unless otherwise indicated. The following levels of statistical significance were used: ^*^, *p* < 0.05; ^**^, *p* ≤ 0.01; ^***^, *p* ≤ 0.001.

## SUPPLEMENTARY MATERIALS







## Abbreviations

BCL6: B-cell lymphoma 6
DLBCL: Diffuse Large B-cell Lymphoma
DOX: Doxycycline
CRISPR: clustered regularly interspaced short palindromic repeats
Cas9: CRISPR-associated protein 9
sgRNA: single guide RNA
