# Supplementary material for: Inducible knock-out of BCL6 in lymphoma cells results in tumor stasis
Source: Oncotarget. 2020 Mar 3;11(9):875–90. doi: 10.18632/oncotarget.27506 (PMC7061739; doi:10.18632/oncotarget.27506)
Supplement: Supplementary file 1 [file oncotarget-11-875-s001.pdf]

Inducible knock-out of BCL6 in lymphoma cells results in tumor stasis

SUPPLEMENTARY MATERIALS

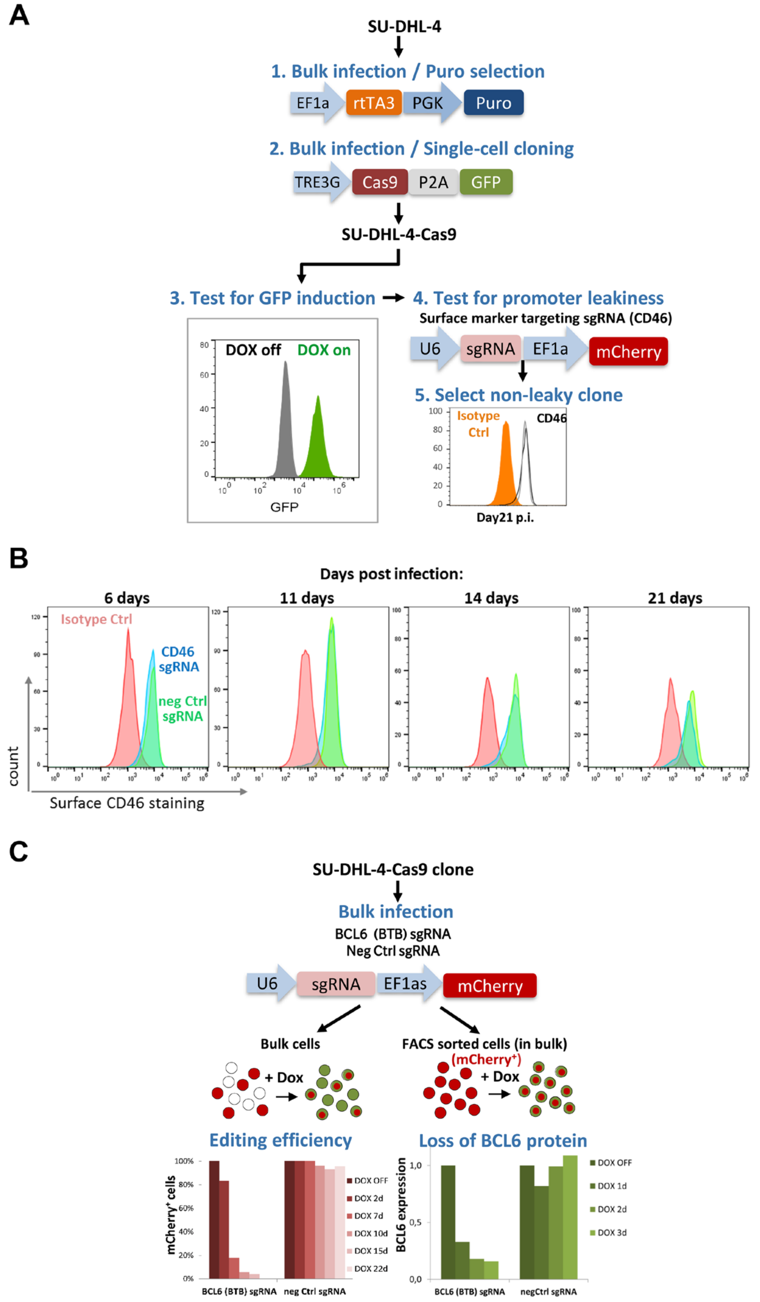

Supplementary Figure 1: Establishment of a DOX-inducible CRISPR/Cas9 System to knock-out BCL6 in DLBCL. (**A**) A DOX-inducible lentiviral CRISPR/Cas9 approach was used to knock-out BCL6 in DLBCL cells. SU-DHL-4 cells were engineered to express the reverse tetracycline-controlled transactivator 3 (rtTA3) and Cas9 under the control of a Tet-On 3G-response element promoter (TRE3G). Single cell clones were tested for GFP induction (reflecting Cas9 expression) and for promoter leakiness. (**B**) Leakiness test of SU-DHL-4 Cas9 clone: SU-DHL-4 Cas9 clones were infected with negative control and CD46 targeting sgRNA. Transduced cells were FACS stained with anti-CD46 antibody and isotype control at day 6, 11, 14, and 21 of culture. The histogram depicts the expression of surface CD46 in CD46 sgRNA infected cells (blue) and negative control infected cells (green). Cells stained with isotype control are depicted in red. (**C**) A selected SU-DHL-4 Cas9 clone was infected with negative control and BCL6 targeting sgRNAs (mCherry^{+}). The editing efficiency was confirmed in a bulk depletion assay (left panel). mCherry^{+} cells were FACS sorted and the loss of BCL6 protein after DOX treatment was investigated using WES protein analysis (right panel).

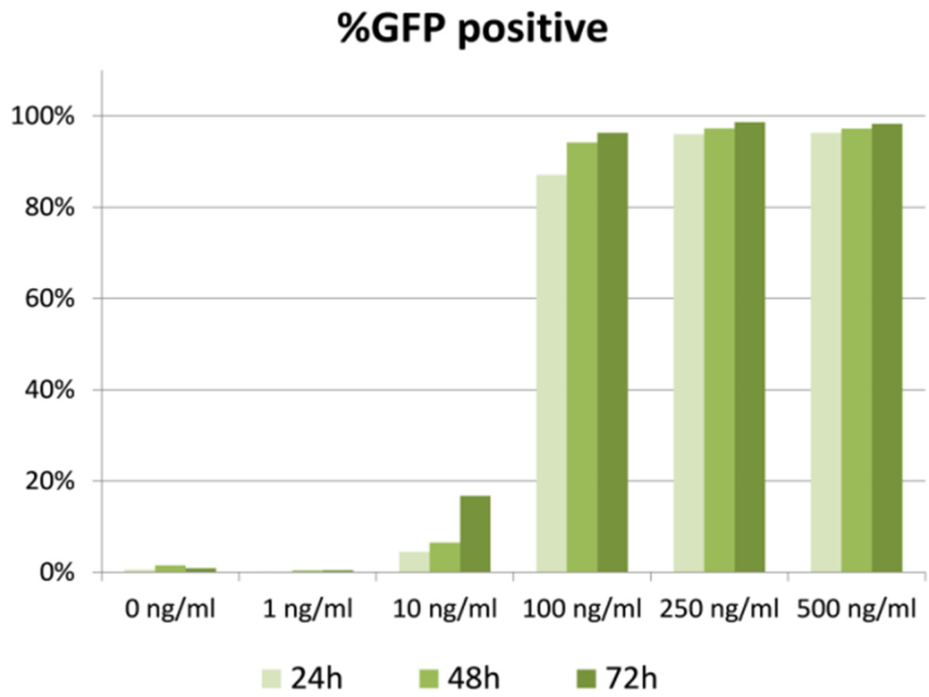

Supplementary Figure 2: GFP induction in SU-DHL-4 Cas9 clone. A selected SU-DHL-4 Cas9 clone was treated with DOX at the indicated concentrations (1–500 ng/ml) for 24 h, 48 h, and 72 h and Cas9 GFP induction was measured using flow cytometry and depicted as percentage of GFP positive cells.

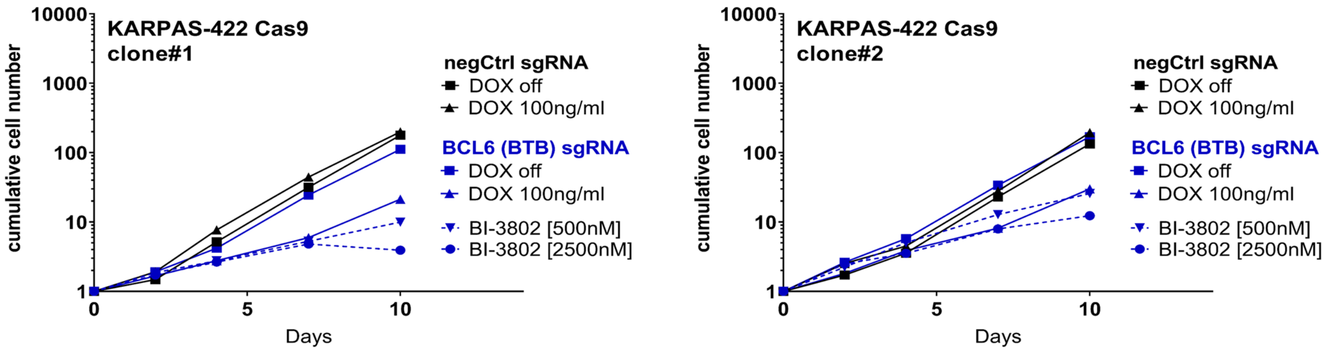

Supplementary Figure 3: Effects of BCL6 knock-out and degradation in KARPAS-422 DLBCL cell line. Long-term proliferation assays with negative control and BCL6 (BTB) sgRNA infected KARPAS-422 Cas9 clones (1+2) after DOX induction (100 ng/ml) and BI-3802 (500 nM, 2500 nM) treatment.

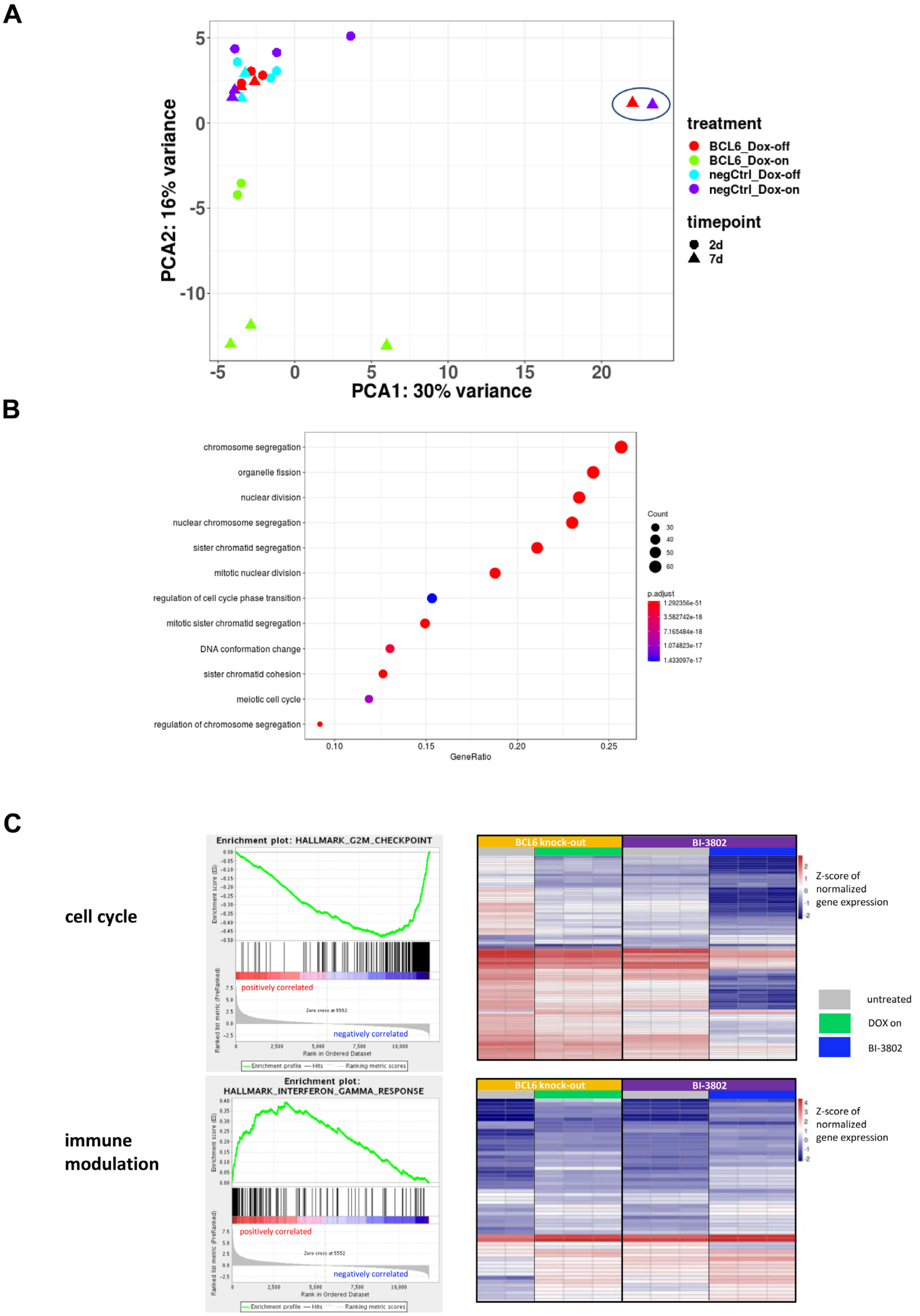

Supplementary Figure 4: Effects of BCL6 knock-out and degradation on gene expression. (**A**) Principal component analysis of the 500 most variable genes comparing all samples from BCL6 knock-out and BI-3802 mediated degradation and their respective controls. The two encircled outlier samples were excluded from all further analysis. (**B**) GO term annotation reveals that genes, which are strongly downregulated upon BCL6 inhibition are predominantly associated with cell cycle and cell division. (**C**) Gene set enrichment examples for cell cycle and immune modulation for the late time point (168 h). Left panels: Enrichment plots, right panels: Expression data (normalized expression for the gene set specific genes (selected for differential expression in BCL6 knock-out (adj. p-value ≤ 0.1, absolute fold change ≥ 2), 2–3 replicates are shown for each condition).

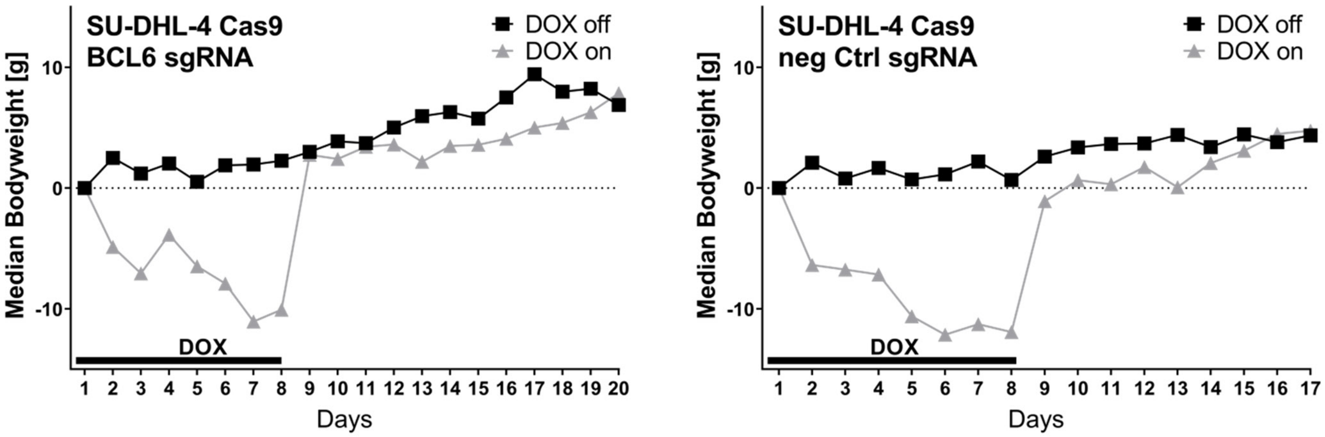

Supplementary Figure 5: Bodyweights of mice during DOX treatment. Bodyweights of tumor-bearing mice were monitored during vehicle (DOX off) and DOX (DOX on) treatment. Duration of DOX treatment is indicated with a black bar. Data are shown as median bodyweight (in g) of mice either carrying BCL6 sgRNA tumors (left, *n* = 10 DOX off, *n* = 10 DOX on) or control tumors (right, *n* = 10 DOX off, *n* = 10 DOX on).

Supplementary Table 1: Number of differentially expressed genes after BCL6 knock-out and degradation in SU-DHL-4 cells

treatment	hours	dn	up
BCL6 knock-out	48 h	12	154
	168 h	271	1037
BI-3802	20 h	10	89
	168 h	1026	656

Supplementary Table 2: List of common up- and down-regulated genes after BCL6 knock-out and BI-3802 mediated BCL6 degradation in SU-DHL-4 cells (related to Figure 5D). See Supplementary Table 2

Supplementary Table 3: Gene set enrichment analysis of differentially regulated genes after BCL6 knock-out and BI-3802 mediated BCL6 degradation in SU-DHL-4 cells. See Supplementary Table 3
